# Publishing trends of journals with manuscripts in PubMed Central: changes from 2008–2009 to 2015–2016

**DOI:** 10.5195/jmla.2018.457

**Published:** 2018-10-01

**Authors:** Lauren Topper, Diane Boehr

**Affiliations:** Contractor, Computercraft Corporation, and PMC Journal Review Program Coordinator, National Library of Medicine, Bethesda, MD; Head, Cataloging and Metadata Management Section, National Library of Medicine, Bethesda, MD

## Abstract

**Objective:**

The National Institutes of Health (NIH) public access policy mandates that all articles containing NIH-funded research must be deposited into PubMed Central (PMC). The aim of this study was to assess publishing trends of journals that were not selected for the National Library of Medicine (NLM) collection but contain NIH-funded articles submitted to PMC in compliance with the public access policy. In addition, the authors investigated the degree to which NIH-funded research is published in journals that NLM does not collect due to concerns with the publishers.

**Methods:**

We analyzed bibliographic data from the NIH Manuscript Submission system for journals that were not selected for the NLM collection from August 2015 to August 2016. Publications (n=738) were analyzed by language, publishing country, publishing format, and subject, and the results were compared to a similar study of 2008–2009 data. In addition, publications were analyzed by whether their publishers are collected by NLM, as determined by transparency and adherence to publishing best practices.

**Results:**

Only a few differences were found between the studies. Most notably, while both studies revealed that most journals were not selected for the NLM collection because they were out of scope (i.e., not biomedical), we noted an increase in 2015–2016 in biomedical journals containing NIH-funded articles that were not added to the collection due to concerns with the publishers.

**Conclusions:**

While the current number of NIH-funded manuscripts being published by publishers that are not collected by NLM remains quite small, we noted a substantial increase between 2008–2009 and 2015–2016.

## INTRODUCTION

The National Library of Medicine (NLM) is the world’s largest biomedical library and is one of the twenty-seven institutes that constitute the National Institutes of Health (NIH). One of NLM’s core missions is to assist in the advancement of medical and related sciences through the collection, dissemination, and exchange of information important to the progress of medicine and health. As part of this mission, NLM launched PubMed Central (PMC) in 2000 as a free, full-text archive of biomedical and life sciences journals that can be searched through PubMed [1].

In 2008, the NIH public access policy was instituted, mandating that all articles describing NIH-funded research must be deposited into PMC, where they would be made freely available to the public within twelve months of publication [2]. NIH-funded authors can comply with this policy in one of two ways: (1) by publishing in journals or publisher programs that have formal agreements with PMC to deposit the version of record of the article directly in PMC, or (2) by depositing the author manuscript version of the article via the NIH Manuscript Submission (NIHMS) system. NIH does not dictate the journals in which their funded authors publish. After the policy took effect, NLM staff found that some manuscripts being submitted through the NIHMS system were published in journals that did not meet the guidelines for inclusion in the NLM collection, as defined by the *NLM Collection Development Manual* [3].

Therefore, in 2011, NLM collected data to investigate publishing trends among these journals and identify if additional titles should be reviewed for the collection [4]. The original study utilized data from the time at which the NIH public access policy was instituted in April 2008 up until October 2009. The results revealed that most NIHMS system manuscripts came from non-biomedical journals considered to be “out of scope” for the collection (i.e., <20% of articles in a journal were biomedical) and that these manuscripts represented interdisciplinary research. These medically related articles enhance the NLM collection without the need for NLM to purchase additional out-of-scope journals.

However, in recent years, there have been reports of publishers producing scientific journals that do not adhere to industry standards and best practices [5–7]. NLM has instituted several review programs designed to select only those journals that follow best practices for the collection. For example, the PMC journal review program, based on scientific and editorial quality, was established in 2014 to evaluate journals that apply to PMC [8].

In addition, when a publisher that is new to NLM applies to include a journal in PMC, an NLM committee first reviews the publisher for conformance with guidelines and best practices, including *Recommendations for the Conduct, Reporting, Editing, and Publication of Scholarly Work in Medical Journals* from the International Committee of Medical Journal Editors (ICMJE) [9] and *Principles of Transparency and Best Practice in Scholarly Publishing,* a joint statement by the Committee on Public Ethics (COPE), Directory of Open Access Journals (DOAJ), World Association of Medical Editors (WAME), and Open Access Scholarly Publishers Association (OASPA) [10]. If a publisher fails to conform to these best practices for publishing, their publications are not selected for the collection [11], and NLM will not consider any journal applications from that publisher for at least three years. After three years, if the publisher submits a journal application to PMC, the NLM review committee will reevaluate the publisher.

Publishers of journals that NLM decides not to collect can include those that are referred to as “predatory publishers” or publishers of “pseudo-journals” and may be identified by several attributes, such as generally misleading business practices, use of aggressive tactics to solicit article submissions, and insufficient peer-review processes. Alternatively, many publishers that NLM does not collect may not be engaging in these practices but simply lack the knowledge or resources to produce a journal of the quality required for the NLM collection. However, if NIH-funded authors publish articles in journals produced by publishers that NLM does not collect, the authors must deposit the manuscript in PMC via the NIHMS system, per the NIH public access policy.

**Figure 1:**
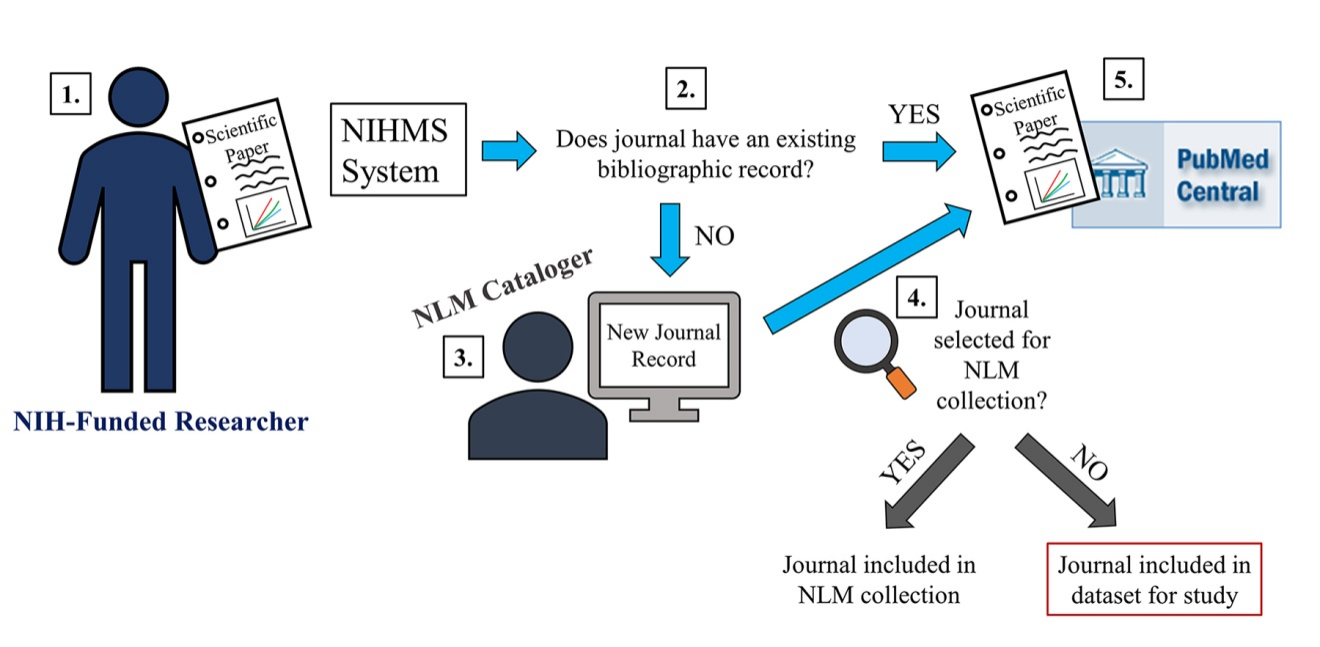
General schematic of bibliographic workflow for articles submitted to PubMed Central (PMC) via the National Institutes of Health Manuscript Submission (NIHMS) system To comply with the NIH public access policy, NIH-funded research published in journals that do not participate in PMC must be manually uploaded by the author through the NIHMS system.The journal in which the article is published must have a bibliographic record in the National Library of Medicine (NLM) catalog before the article can be added to PMC. If a record exists, the article can be directly deposited in PMC (step 5) without any further steps.If a record does not exist, the submission is routed to an NLM cataloger who creates a record for the journal.In addition, the “new to NLM” journal is evaluated for potential inclusion in the NLM collection. Journals that are evaluated but not selected for the NLM collection (red outline) constitute the dataset used for this analysis and for the 2008–2009 dataset [4].Regardless of the outcome of the journal evaluation, once the journal has a record in the NLM bibliographic database, the article is deposited in PMC. Bibliographic records in the NLM catalog contain information about the journal, including whether the journal is part of the NLM collection or any of NLM’s literature databases, such as MEDLINE and PMC. NLM’s bibliographic records can be searched via LocatorPlus or the NLM Catalog.

The NLM workflow requires that a bibliographic record for a journal title exists in the NLM online catalog before articles from that journal can be added to PMC (Figure 1). Most articles submitted through the NIHMS system already have existing journal records and can flow directly into PMC as they are submitted. However, the NIHMS system routes journals that lack NLM bibliographic records but contain NIH-funded research to catalogers so that new records are created. The journal is then evaluated by NLM selectors for inclusion in the NLM collection, and if the journal is not selected, it is indicated in the record.

In this study, the authors obtained bibliographic data generated from the NIHMS system (i.e., journals newly added to the NLM bibliographic database) from August 2015 to August 2016 and performed an analysis of the materials from this dataset that were not selected for the NLM collection. Our study had two primary aims. The first aim was to assess how publishing trends of author-submitted manuscripts have evolved since 2008–2009 by comparing our findings against those of the original study [4]. The second aim was to investigate the degree to which NIH-funded research is being published by publishers that the NLM has decided not to collect.

## METHODS

### Generation of the National Institutes of Health Manuscript Submission (NIHMS) dataset

The list of journals to be reviewed was generated from the National Center for Biotechnology Information (NCBI) Publisher Portal. This internal-to-NLM system tracks all requests for new journal records that need to be created for submitted manuscripts because no record already exists in the NLM bibliographic database. The list of requests originating from the NIHMS system was then compared to the bibliographic records that had been created and limited to those records for which the final status was “Not Our Catalog/Collection” (NOC), as indicated in the MARC 999 field with a value of NOC, to ensure the journals included in the dataset had not been selected for collection by NLM.

The NLM collection not only includes journals in PubMed (comprising primarily citations from journals selected for PMC and MEDLINE, NLM’s journal citation database), but also extends to all titles that NLM purchases or provides access to. In this study, only new bibliographic entries for publications that contained one or more NIH-funded, peer-reviewed author manuscripts (generally journals, but also some monographs containing conference publications) and that were not selected for the NLM collection were included (Figure 1), for a total of 738 publications. While we used the same methodology to obtain our data, this is a somewhat larger set than the 571 publications reviewed for the 2011 study, despite the fact that the 2011 study used a slightly longer time frame (roughly 18 months) and likely reflects the overall growth in NIHMS submissions since the NIH public access policy was instituted. The data were analyzed between December 2016 and January 2017.

### Comparison of journals by topic

To allow direct comparison, publications were grouped by the same fifteen subject categories that were utilized in the original study in 2011 [4], which were constructed according to the Library of Congress Classification [12]. However, a new subject area “broad” was also included, which was created to accommodate journals that accept all, or multiple, scientific fields. Therefore, sixteen categories were used: agriculture, anthropology, broad, chemistry, computer science, economic theory/finance, education, engineering, fine arts/language and literature, library science, mathematics, medicine, natural history/biology/zoology, physics, political science/law, and psychology.

### Comparison of journals by publisher status

Each publisher was evaluated based on whether it had been assessed by an NLM review committee and whether the committee had decided to collect content from that publisher. Accordingly, publishers were classified into three categories. The first category was “collected or not reviewed,” which denoted publishers that had been reviewed by an NLM committee and found to meet accepted standards as well as publishers that have never been reviewed by a committee. NLM began performing publisher reviews in 2016 and typically only reviews either publishers that are new to NLM or those that are established but about which concerns have been raised. As such, many large and long-established publishers have not undergone a publisher review but are collected by NLM. Since journals from these publishers accounted for a significant portion of the data, we felt that it would be a more accurate representation to group publishers that had never undergone a review with publishers that had been formally reviewed and the committee had decided to collect. The second category was “not collected,” which denoted publishers that had been reviewed by an NLM committee and found not to meet accepted standards. The third category was “under review,” which referred to journals that were being reviewed by the committee at the time the data were analyzed.

## RESULTS

### Aim 1: General characteristics and publishing trends of author-submitted manuscripts

Publications were first classified by the language in which they were published. The overwhelming majority were found to be in English (95.9%), in agreement with the original study of 2008–2009 data, which reported that 98.5% were in English [4]. Moreover, we found that an additional 2.5% of publications were published in more than 1 language, which always included English. The next most common languages were French (0.05%) and Spanish (0.02%).

**Figure 2:**
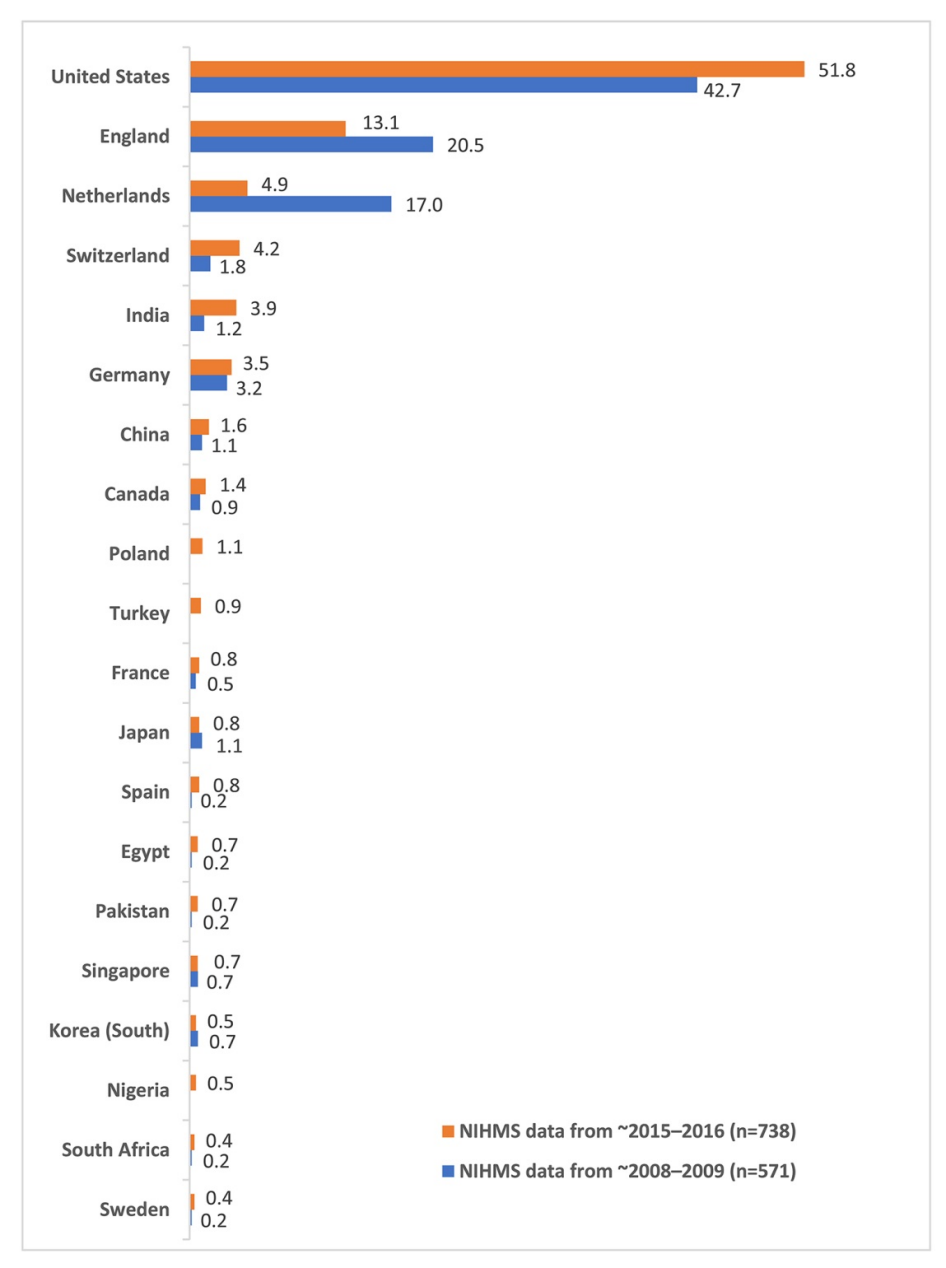
Percentage of publications in the NIHMS datasets by country The top 20 countries according to ~2015–2016 NIHMS data are shown and compared to the corresponding percentage of publications from ~2008–2009 data. When no percentage is reported for a country, the number of publications is 0. ~2008–2009 NIHMS data were taken from a previously published study [4].

Next, publications were classified by the location (i.e., country) of the publisher (Figure 2). Most were published in the United States, followed by England, the Netherlands, and Switzerland. Again, these findings were similar to those reported in 2011 (Figure 2), which showed the following top 4 countries: United States, England, the Netherlands, and New Zealand (4%; not shown in Figure 2, as New Zealand had only 1 publication in the 2015–2016 dataset). The most notable trends from 2008–2009 to the present were an increase in the number of publications published in the United States and India and a decrease in the number of publications published in England, the Netherlands, and New Zealand.

**Figure 3:**
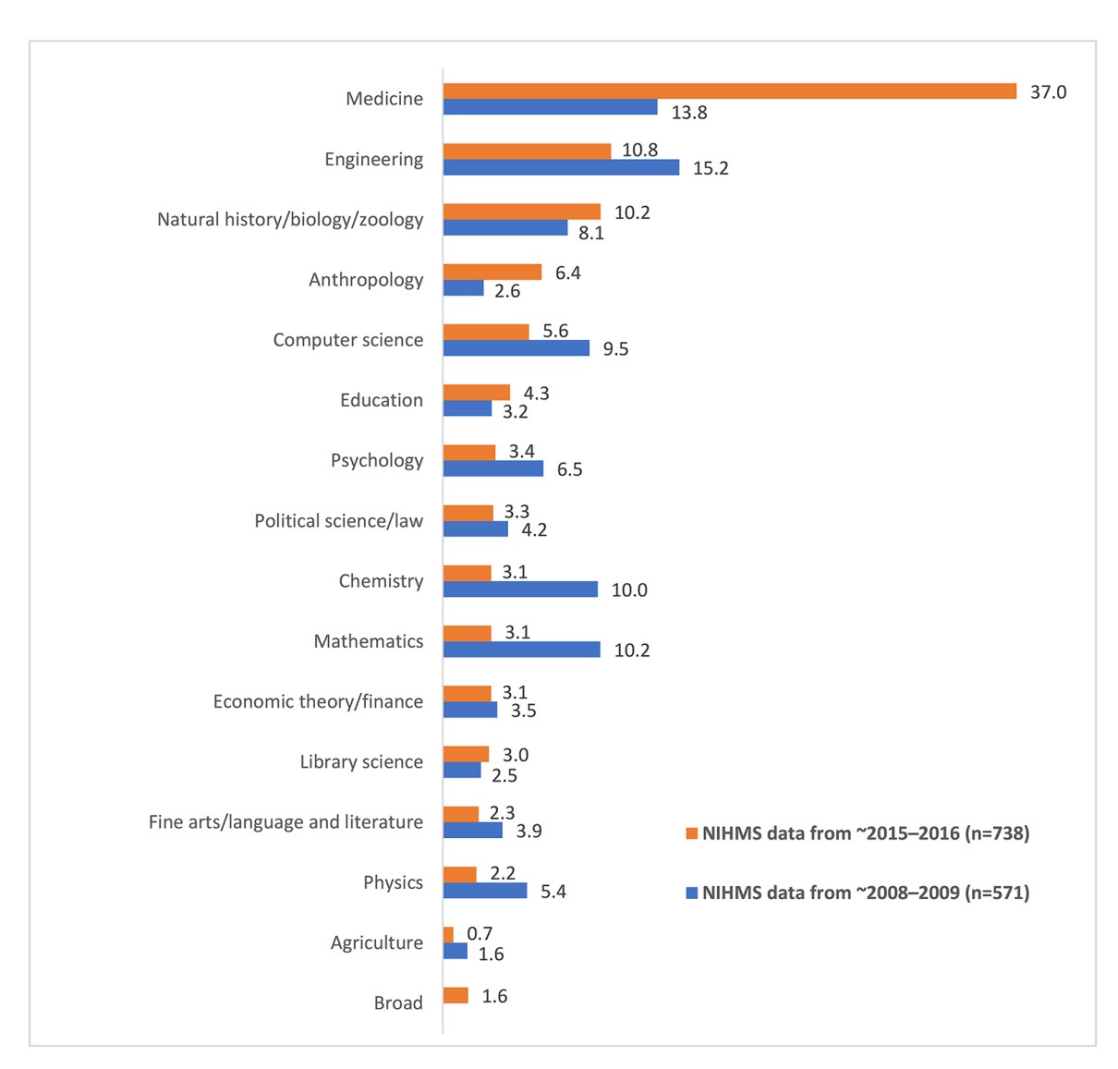
Percentage of publications in the NIHMS datasets by subject ~2008–2009 NIHMS data are taken from a previously published study [4], in which “broad” was not used as a category.

Publications were also analyzed by subject (Figure 3). The most common classification was “medicine,” with “engineering” and “natural history/biology/zoology” the next most prominent subjects. These results are in contrast with the 2008–2009 data (Figure 3), which showed “engineering” as the most popular subject area, followed by “medicine” and “mathematics” [4]. In addition, in our current analysis, 12 publications were classified as “broad.” Of these, 5 were journals with a stated scope that included all fields of science and technology, and another 5 were journals that included a very wide range of subject areas, not limited to science (e.g., science, business, humanities, law). Of the final 2 publications, one was a published version of conference proceedings, and the other was a journal devoted to research on publishing ethics.

### Comparison by type of publication

The NIH public access policy requires all peer-reviewed manuscripts accepted for publication in a journal that are the product of direct NIH support to be submitted to PMC [2]. Book chapters are excluded. However, in 2014, recognizing that a notable amount of peer-reviewed, NIH-funded research was being published as part of engineering and computer science conferences, NLM agreed to make an exception for conference publications, where very similar proceedings from different conferences are variously treated in library catalogs as monographs or serials, as permitted by the Cooperative Serials Program (CONSER) of the Program for Cooperative Cataloging guidelines [13]. Because NLM uses existing OCLC copy whenever possible to create its bibliographic records, we found instances where only monographic records are available for conference publications. In this study, 72 publications (10%) were identified as conference proceedings. Of those, 49 (68%) were in publications cataloged as serials and 23 (32%) were in publications cataloged as monographs.

### Aim 2: Assessment of NIHMS system manuscripts by publisher

**Figure 4:**
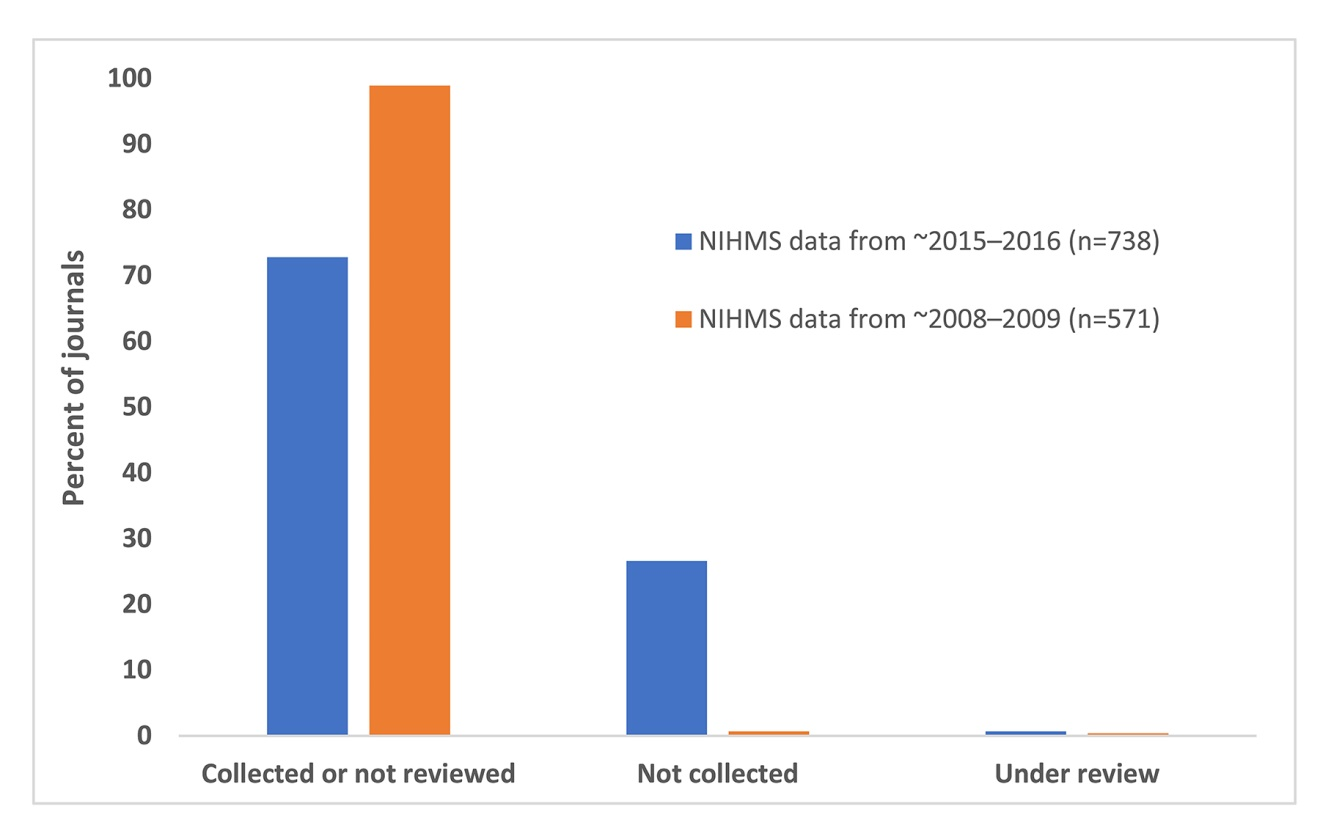
Percentage of publications in the NIHMS datasets that were produced by publishers NLM does not collect ~2008–2009 NIHMS data are taken from a previously published study [4] and were reanalyzed based on current publisher status.

Of the 738 total publications, 196 were from publishers that were not collected by NLM and 537 were from publishers that were either collected or had not been reviewed (Figure 4). An additional 5 publications were from publishers classified as under review. Nearly half of the publications classified as “medicine” in the subject area analysis were not collected by NLM (49%; 134 of 273 total), whereas a smaller proportion of publications classified as “broad” were not collected by NLM (17%; 2 of 12 total).

A search of PubMed revealed that while most publications from publishers not collected by NLM contained only the NIH-funded manuscript for which the bibliographic data were created (133 of 196), 47 contained between 2 and 4 manuscripts, and 1 publication contained 7. The most prevalent publisher that was not collected by NLM was the OMICS Group, which was responsible for 49 manuscripts in a total of 37 journals. The second most prevalent publisher that was not collected by NLM was Insight Medical Publishing with 14 journals, followed by Scientific Research Publishing with 12 journals.

Because much of the concern surrounding publishers that do not follow current best publishing practices stems from their recent upsurge, we reanalyzed the original dataset from the 2011 study [4] to determine how many journals during that time frame came from publishers that were not currently collected by NLM. We found that of the 571 total publications, only 4 were from publishers that NLM did not collect, and 2 were from publishers that were under review (Figure 4).

## DISCUSSION

Over the last few years, PMC has seen tremendous growth, increasing from approximately 2,000,000 articles in 2010 to over 4,000,000 articles in 2016 [1]. In addition, more than 100,000 NIH-funded papers are deposited in PMC annually following the institution of the NIH public access policy (based on a search of PubMed data). Over half of these articles are deposited in PMC via the NIHMS system as author manuscripts rather than through journals or publisher programs that have formal agreements with PMC. Here, we obtained bibliographic data generated from the NIHMS system to analyze the publishing trends of author-submitted manuscripts for journals that were not already part of the NLM collection and compared them to data from 2008–2009 [4].

### Aim 1: General characteristics and publishing trends of author-submitted manuscripts

The most noticeable change in publishing trends between this study and the one conducted in 2011 was with respect to publication topics. Specifically, there was a stark increase in the percentage of NIHMS-submitted manuscripts from journals classified as “medicine.” The previous study concluded that most journals containing NIHMS system manuscripts were not selected for the NLM collection due to scope, which may still be the case; however, a much larger number of new journals that were not selected are classified as “medicine” and should therefore be within scope. It is possible that the increased number of author-submitted manuscripts from medical publications indicates an overall increase in medical research and a corresponding increase in the number of new medical journals. As an example, between 2008 and 2016, more than 300 journals and over 6.5 million citations were added to MEDLINE [14]. Conversely, it could also reflect a rise in the number of publications with questionable publishing practices in the field. A recent study by Shen and Bjork indicated an increase in the prevalence of questionable publishers, with biomedicine being one of the most affected fields [15]. While their analysis was limited to open access publishers, their findings are consistent with our observation regarding the relatively high percentage of publishers that NLM does not collect among journals classified as “medicine.”

In addition, we did note some changes in the locations of the publishers producing these journals. However, it is important to note that these data were typically derived from the publications’ websites. Many publishers have multiple locations, and only the first is recorded in bibliographic records. How the same publisher presents their location in different publications can, therefore, skew the results. It is also possible that some data are inaccurate, as some journals have been reported to falsely advertise their locations [6, 11]. Therefore, it is difficult to draw clear conclusions from these data.

Finally, the addition of peer-reviewed papers from conference proceedings published as monographs allowed us to include potentially valuable articles in PMC that were previously excluded and removed a dichotomy that was difficult to explain or justify to authors [13].

### Aim 2: Assessment of NIHMS system manuscripts by publisher

The results of our study revealed that several journals that were added as new bibliographic entries following author submission to the NIHMS system were not selected by NLM due to concerns with the business practices and transparency of the publishers. While we found that most journals that were not selected were likely out of scope for the collection, as was the case in the 2011 study [4], the stark increase in the number of publications that NLM does not collect over time indicates that publisher status is playing a much more prominent role in determining selection.

As a limitation, it should be reiterated that these data only reflect journals that were new to NLM during this time frame. The NIHMS system receives a vast number of submissions, and approximately 92% of those articles are published in journals that participate in MEDLINE (the NLM journal citation database, which has its own review program) and have, therefore, already been recommended by an NLM committee [16]. In the same time frame as this study (August 2015–August 2016), over 65,000 manuscripts were processed through the NIHMS system from 5,689 different journals. As such, the overall number of NIH-funded author manuscripts that are published in questionable journals is quite small, although future work using a more comprehensive dataset is required to obtain an exact measurement.

Interestingly, we also noted that the most prevalent publisher that NLM did not collect among our dataset was the OMICS Group. At present, the OMICS Group is being sued by the Federal Trade Commission (FTC) [5]. According to the FTC website, OMICS is charged with making false claims, as well as failing to disclose significant publishing fees until after articles have been accepted and then refusing to allow authors to withdraw their articles. Many of the charges filed against OMICS represent practices that are considered characteristic of predatory publishers [6].

In general, a common hallmark of predatory publishers is a lack of transparency, such as a publisher that attempts to mislead readers about its location or what members constitute its editorial board. In an example of the latter, instances have been reported in which prominent scientists are listed as editorial board members or reviewers who have never agreed to be on the board or perhaps never even heard of the journal [5–7]. A recent “sting” investigation published in *Nature* revealed that many of these journals appoint unqualified scientists to editorial boards without any screening process [17]. Finally, many of these publishers use high-pressure tactics to attract submissions, such as spamming scientists’ email inboxes [6]. However, many of the publishers that NLM does not collect, including those identified in this study, may not be deliberately engaging in misleading and predatory practices but may simply lack the knowledge or resources to produce a journal of the caliber required for the NLM collection.

It is unclear why scientists choose to publish in questionable journals. In many cases, they are likely unaware of the journal’s nature. One study revealed that most authors who publish in questionable journals are young, inexperienced researchers from developing countries [18]. However, our results indicate that this problem exists among US-based scientists of a caliber high enough to win or be supported by NIH grants. Moreover, a recent study of articles published in predatory journals showed that while only 17% of articles acknowledged a funding source, the most common acknowledged funder was NIH [19]. This is likely due to the large number of research studies that NIH funds, as the largest funder of biomedical research in the world [20]. Unfortunately, the inclusion of these individual, NIH-funded articles in PMC has resulted in confusion and sometimes been mistaken for inclusion in PMC at the journal level [21].

In an important step toward reducing the number of NIH-funded manuscripts being published in journals from publishers with questionable practices, NIH issued a guide notice in November 2017 that encourages its funded authors to publish in reputable journals [22]. Moreover, the notice provides tips to help researchers identify questionable publishers and journals, including referencing the Think Check Submit resource [23], and calls on NIH stakeholders such as librarians to help guide authors. In particular, librarians can play an important role by informing patrons at their institutions about the realities (both positive and negative) of scholarly publishing. Librarians can also direct their researchers to information regarding how to identify publishers and journals that are engaged in best publishing practices [24].

However, it is also possible that some authors choose to publish in journals from questionable publishers knowingly in an effort to build their publication records, as publishing can be critical for career advancement [25]. In this case, it may be necessary for funding agencies and academic institutions to become involved, such as by examining the journals in which their staff and prospective employees publish and by excluding journals of questionable quality from curricula vitae when making hiring and promotional decisions.
